# Cryopreservation of Parathyroid Glands

**DOI:** 10.1155/2010/829540

**Published:** 2010-12-08

**Authors:** Marlon A. Guerrero

**Affiliations:** Department of Surgery, The University of Arizona, 1501 N. Campbell Avenue, Tucson, AZ 85724, USA

## Abstract

The risk of permanent hypoparathyroidism following thyroid and parathyroid surgery is around 1% in the hands of experienced endocrine surgeons. Although this complication is rare, rendering a patient permanently aparathyroid has significant consequences on the health and quality of life of the patient. Immediate autotransplantation of parathyroid glands that are injured or unintentionally removed offers the best possibility of graft viability and functionality. However, since the majority of cases of hypoparathyroidism are transient, immediate autotransplantation can complicate postoperative surveillance in certain patients, especially those with primary hyperparathyroidism. Cryopreservation of parathyroid tissue is an alternate technique that was developed to treat patients with permanent hypoparathyroidism. This method allows for parathyroid tissue to be stored and then autotransplanted in a delayed fashion once permanent hypoparathyroidism is confirmed. This article provides a contemporary review on cryopreservation of parathyroid tissue and its current role in thyroid and parathyroid surgery.

## 1. Introduction

Permanent postoperative hypoparathyroidism results from the unintentional removal or injury of parathyroid glands during thyroid and parathyroid surgery. Permanent hypoparathyroidism is defined as persistent hypocalcemia requiring calcium and vitamin D supplementation 6 months after surgery [1]. The development of hypoparathyroidism is contingent on the type of operation performed. The risk is nominal during a minimally invasive parathyroidectomy for a single adenoma but is greatest after a subtotal or total parathyroidectomy, thyroid resection and nodal dissection for large and extensive thyroid cancers, and reoperative neck operations [1]. Even though the risk of transient hypocalcemia can be high during an extensive neck dissection, the permanent hypoparathyroidism rate is typically around 1% in the hands of experienced endocrine surgeons at high-volume centers. 

The accidental onset of permanent hypoparathyroidism can be agonizing for the patient and the clinician alike. For the patient, its negative impact includes a reduced quality of life, expensive lifelong medication supplementation, frequent laboratory testing, and the potential for frequent hospital admissions. In addition, the persistent absence of parathyroid hormone (PTH) has long-term systemic effects on the body, such as the development of osteoporosis (due to the decreased function of osteocytes), premature cataracts, cardiac dysfunction, and neurologic dysfunction [1, 2]. 

In the late 1960s and 1970s, several new techniques were introduced in an attempt to prevent the detrimental health and social consequences of permanent hypoparathyroidism. For example, intraoperative autotransplantation of parathyroid tissue into the sternocleidomastoid muscle or the brachioradialis muscle was recommended. However, not all patients at risk of permanent hypoparathyroidism actually develop it, nor do all patients require an immediate autotransplant. In fact, an unnecessary autotransplant, if performed during parathyroid surgery, could result in persistent hyperparathyroidism. Additionally, the autograft could become autonomously hyperfunctional, posing a diagnostic and treatment dilemma in the future; Wells et al. overcame that limitation and transformed our approach to prevent hypoparathyroidism by introducing autotransplantation of cryopreserved parathyroid tissue [3]. 

Cryopreservation permits parathyroid tissue to be stored for potential reimplantation, thereby avoiding the risk of needlessly implanting parathyroid tissue during initial surgery. The clinician is able to accurately determine whether any residual parathyroid tissue will recover function or whether a delayed autotransplant will be needed [4]. Most patients with postoperative hypoparathyroidism have the condition only transiently. The disadvantages of cryopreservation of parathyroid tissue include the potential of graft failure and the risk of graft-dependent hypercalcemia [5]. This paper provides an up-to-date, comprehensive review of the cryopreservation of parathyroid tissue and its current role in thyroid and parathyroid surgery.

## 2. Indications for Cryopreservation

### 2.1. Initial Neck Operations

The clear indication for an autotransplant of cryopreserved parathyroid tissue is permanent postoperative hypoparathyroidism. In the hands of experienced endocrine surgeons, the risk of permanent hypocalcemia is ≤1% during initial neck operations. The risk is particularly low in patients with sporadic primary hyperparathyroidism (PHPT), most of whom have a single parathyroid adenoma. 

Nonetheless, certain patients have a higher risk of developing permanent hypoparathyroidism after their initial neck surgery (Table 1). Most commonly at risk are patients with multigland parathyroid hyperplasia, especially those with familial primary hyperparathyroidism [6]. Such patients may undergo either a subtotal (3.5-gland) parathyroidectomy or a total parathyroidectomy with an immediate autotransplant. Both a subtotal parathyroidectomy and a total parathyroidectomy can result in aparathyroidism, so cryopreservation of parathyroid tissue is recommended at the time of the initial surgery [6]. In addition, it has been reported that the use of intraoperative parathyroid hormone (IOPTH) monitoring during parathyroid surgery can accurately predict patients at risk of developing postoperative hypocalcemia. A drop of >80% of IOPTH at 10 minutes is a significant factor for postoperative hypocalcemia [7]. Therefore, cryopreservation of parathyroid tissue should be considered during parathyroid surgery when the IOPTH drop >80%.

Patients with end-stage renal disease are at high risk of developing secondary (SHPT) and tertiary (THPT) hyperparathyroidism. Such patients have persistent stimulation of the parathyroid glands secondary to abnormalities in the metabolism of calcium, phosphorus, and vitamin D, resulting in multigland parathyroid hyperplasia. The choice of operation for such patients can be problematic. They have an inherently high risk of disease recurrence if a subtotal parathyroidectomy is performed but a high risk of permanent hypoparathyroidism if a total parathyroidectomy and an immediate autotransplant are performed [8]. Moreover, such patients may require multiple operations; to prevent aparathyroidism as a consequence of the initial or subsequent operations, cryopreservation of parathyroid tissue is recommended.

Surgical treatment of patients with thyroid disease can also lead to permanent hypoparathyroidism. The risk of aparathyroidism is nominal with only a thyroidectomy but increases with more extensive surgical resections. Routine central neck dissections for thyroid cancer have a complication rate of permanent hypoparathyroidism of up to 14% [9]. Given such risks, the role of prophylactic central neck dissections for papillary thyroid cancer continues to be debated. An immediate intraoperative autotransplant is preferred during a neck dissection for thyroid cancer, yet cryopreservation of parathyroid tissue fragments is also warranted given the multiple operations that may be required in the future. Subsequent operations increase the risk of permanent aparathyroidism, an outcome that cryopreservation of parathyroid tissue may prevent.

### 2.2. Redo Neck Operations

In contrast to initial neck operations, the risk of permanent hypocalcemia after a redo neck operation is not minuscule, but rather as high as 30% [6, 10]. The risk of aparathyroidism is higher during a redo neck operation because the viability of the remaining parathyroid glands cannot be adequately determined. During the initial surgery, parathyroid glands left in situ may have unknowingly been devascularized or damaged. During a redo neck operation, removal or inadvertent injury of any remaining functional gland may result in permanent hypoparathyroidism. This scenario can be particularly problematic in patients who develop hyperparathyroidism after previously undergoing a thyroidectomy; during a redo neck operation, any parathyroid gland removed must be assumed to be the last viable parathyroid gland and should be autotransplanted immediately to optimize transplantation success. A fragment of the parathyroid tissue may be cryopreserved for a possible delayed autotransplantation if future neck reoperations result in aparathyroidism. 

Other common reasons for parathyroid reoperations include persistent hyperparathyroidism (hypercalcemia <6 months after the initial surgery) and recurrent hyperparathyroidism (hypercalcemia >6 months after the initial surgery). Operative success for redo parathyroid surgery is <90% and is fraught with an 18% rate of causing permanent hypoparathyroidism [11]. Immediate autotransplantation of parathyroid tissue to prevent hypoparathyroidism impedes determination of surgical outcome, which is unpredictable during redo parathyroid surgery [10]. Accurately assessing the number of parathyroid glands previously resected, or the viability of the remaining parathyroid glands, is difficult. Cryopreservation of parathyroid tissue, rather than an immediate autotransplant, allows the surgeon to appropriately predict surgical outcome, the functionality of any remaining parathyroid tissue, and the need for a delayed parathyroid autotransplant.

Operative failure and disease recurrence are infrequent in patients with sporadic PHPT; reoperations are more commonly needed in those with SHPT and THPT [8]. Up to 15% of patients with SHPT and THPT tend to have more than four parathyroid glands [12], increasing the risk of operative failure. Cryopreservation of their parathyroid tissue is especially important, because the long-term renal effect of prolonged SHPT and THPT predisposes patients to transient hypocalcemia from bone hunger. Before any parathyroid autotransplant, it is critical to differentiate transient from permanent hypoparathyroidism. Similarly, patients with thyroid cancer who require repeating operations are at risk of hypoparathyroidism, because their remaining parathyroid gland function cannot properly be assessed. Furthermore, if parathyroid glands are immediately autotransplanted into the neck muscles during the initial thyroid surgery, the function of those glands cannot be properly evaluated [10]. During reoperations for thyroid cancer, a portion of any inadvertently removed or devascularized parathyroid glands should be sent for cryopreservation before any immediate autotransplant. Patients who develop aparathyroidism during subsequent operations might later benefit from a delayed autotransplant of the cryopreserved parathyroid remnant.

## 3. Cryopreservation Techniques

### 3.1. Tissue Preparation

Intraoperative preparation of parathyroid tissue is relatively uniform. The parathyroid tissue removed during surgery is dissected into 30 to 40 pieces of 1 × 1 × 1 mm. The pieces are then placed into a sterile vial containing ice-chilled saline. The vial is then transported. The supernatant is decanted; about 10 tissue fragments are transferred into each sterile freezing vial to be resuspended in the freezing media.

### 3.2. Freezing Media

The cryopreservation process is vital to preserving graft functionality. A crucial component is the freezing medium used during the freezing process. Several media have been proposed in the literature since the initial report by Wells et al. [13]. The typical freezing medium contains Roswell Park Memorial Institute (RPMI) 1640 solution [2, 4, 6, 10, 13–16]. Most institutions use an 80% RPMI 1640 solution [2, 4, 10, 13], but some use either a 60% RPMI solution 1640 [15] or no RPMI 1640 solution [17]. 

In addition, some authors recommend supplementing the RPMI 1640 solution with 2 mM of glutamine [11, 16] and 5  *μ*g/mL of penicillin streptomycin [16] or 50  *μ*g/mL of gentamicin [11]. Dimethyl sulfoxide (DMSO) in a 10% concentration is added as a cytoplasmic stabilizer [2, 4, 10, 11, 15]. Other reported concentrations of DMSO range from 7.5% to 20% [16, 18]. The last component of the storage medium is either a 10% to 30% autologous serum [2, 10, 11, 15, 16] or a 10% to 20% fetal bovine serum [4, 11]. When the RPMI 1640 solution is excluded, a 90% fetal bovine serum is used [17]. 

### 3.3. Freezing Process

The goal of the freezing process is to preserve cellular function by maintaining cellular integrity through the temperature change. To accomplish that goal, the vials are cooled slowly, before being transferred into long-term storage. A cooling technique developed at the Mayo Clinic entails placing the vials in an ice chest filled with dry ice prechilled to −55°C to −60°C for 1 hour to allow cooling by −1°C per minute [11]. Other authors recommend placing the vials in a −60°C ethyl alcohol bath [10] or in either a −70°C [15] or a −80°C [17] freezer overnight. The vials can also be placed in a programmable freezer and cooled by −1°C per minute until a temperature of −80°C is reached [16]. Once the vials are cooled, they are transferred into a liquid nitrogen freezer and stored at any of several recommended temperatures: −170°C [15, 16], −180°C [4, 17], −190°C [10], or −196°C [2]. 

## 4. Autotransplant Techniques

### 4.1. Thawing

The vials containing the parathyroid tissue designated for delayed autotransplantation are removed from the liquid nitrogen freezer and placed in a warm water bath. The vials are shaken at 37°C [11, 17] to 42°C [10] until the parathyroid tissue fragments are thawed. The fragments are washed in RPMI 1640 solution at 37°C three times [10, 11]. Other authors recommend rinsing the fragments five times in 1 mL of RPMI 1640 solution with a 20% autologous serum [2]. The RPMI wash is performed to rinse the DMSO, which is toxic to cells at room temperature.

### 4.2. Reimplantation

The patient's nondominant forearm is chosen for the parathyroid tissue reimplantation under local anesthesia. A longitudinal incision is made on the forearm. Dissection is continued until a flexor muscle, preferably the brachioradialis muscle, is exposed. Bluntly, pockets are created in the brachioradialis muscle. One to three parathyroid graft fragments are transplanted into separate muscle pockets [13]. To maximize the chances of graft function, a total of 20 to 40 fragments are autotransplanted [2, 10]. Care must be taken to not cause an intramuscular hematoma, which can compromise graft function. The pockets are closed with nonabsorbable sutures and marked with a metal clip.

## 5. Functionality Testing Posttransplant

Success posttransplant is determined by the functionality of the cryopreserved parathyroid tissue graft. Functionality is determined not only clinically but also biochemically (by sampling blood from the grafted and nongrafted antecubital veins, to determine the PTH level at both sites). The parathyroid graft is reported as fully functional, partially functional, or nonfunctional (Table 2). 

Clinically, the graft is considered fully functional when the patient remains eucalcemic and asymptomatic—after all the calcium and vitamin D supplementation has been discontinued. Complete independence from exogenous supplementation is considered the best evidence of graft function [10]. A graft is also considered fully functional when the PTH ratio between the grafted and nongrafted arms is greater than 1.5—again, after all the calcium and vitamin D supplementation has been discontinued [11]. Others recommend a PTH ratio of greater than 2 [10]. 

The graft is considered partially functional when the patient continues to require calcium supplementation, with or without vitamin D supplementation, with a PTH ratio of greater than 1.5 [11]. 

The graft is considered nonfunctional when the patient is hypocalcemic and requires calcium supplementation, with or without vitamin D supplementation, with a PTH ratio of less than 1.5.

In renal patients, the functionality of the parathyroid graft is determined by their PTH levels, independent of calcium and vitamin D supplementation (since most renal patients require supplementation) (Table 2) [18]. 

## 6. Effects of Cryopreservation

The potential benefits of cryopreservation are limited by the reduced functionality of cryopreserved grafts, as compared with immediately autotransplanted grafts [11]. Cryopreserved grafts retain functionality in <70% of patients; fresh autografts >90% [2, 15]. The cryopreservation process may induce cellular necrosis and impair cellular viability and, ultimately, cellular function. The key question is this: does cell viability determine cell function? Earlier studies found no difference in cell viability and secretory capacity between fresh and cryopreserved parathyroid tissue grafts [6, 19]. Yet other studies demonstrated that, even though the percentage of viable cells did not necessarily differ between fresh and cryopreserved tissue, cryopreservation decreased the total number of live cells by >70% [4]. That effect on live cell yield was the same whether the parathyroid tissue was frozen as tissue fragments or as dispersed cells [4]. 

Recently, other authors reported that the cryopreservation process decreased total cell viability [15] and that decreased cell viability was associated with increased storage time [17]. Functionality also depended on storage time [15]. Both viability and function were drastically reduced with a storage time of 22 months [15, 17]. To counterbalance the effects of cellular necrosis, some authors routinely perform histologic examination of the cryopreserved tissue and autotransplant parathyroid tissue according to the percentage of necrotic cells [2]. In addition, cryopreserved parathyroid tissue should be utilized as soon as it is deemed necessary, since a longer storage time limits delayed autotransplant success.

## 7. Conclusion

The devastating effect of permanent aparathyroidism has been, for the most part, ameliorated by parathyroid gland cryopreservation and delayed autotransplants. Cryopreservation is an extremely valuable tool that is exceedingly useful in parathyroid surgery. An immediate autotransplant is preferred during thyroid surgery, yet cryopreservation of a portion of the parathyroid tissue can also greatly help patients at high risk of undergoing further surgery. Cryopreservation allows the clinician to make appropriate decisions regarding the status of the remaining parathyroid glands. Such enhanced decision making is important because most patients undergoing parathyroid and thyroid surgery experience only transient hypocalcemia. Differentiating between transient and permanent hypocalcemia is critical, especially after a parathyroidectomy when hypocalcemia may result from bone hunger, rather than insufficiency of PTH. Cryopreservation facilitates appropriate surgical and clinical decisions, prevents unnecessary immediate parathyroid autotransplants, and offers a chance to cure aparathyroidism.

## Figures and Tables

**Table 1:** Indications for parathyroid cryopreservation.

Initial neck operations	Redo neck operations
Multigland parathyroid hyperplasia	Parathyroidectomy after thyroidectomy
– Familial primary hyperparathyroidism (PHPT)	Persistent hyperparathyroidism
– Secondary hyperparathyroidism (SHPT)	Recurrent hyperparathyroidism
– Tertiary hyperparathyroidism (THPT)	Redo central neck dissection for thyroid cancer
Total thyroidectomy with central neck dissection	

**Table 2:** Definitions of parathyroid graft functionality.

Fully functional	*Asymptomatic nonrenal patients*
	– Off calcium and vitamin D supplementation
	– Eucalcemic with normal PTH levels and/or
	grafted-to-nongrafted arm, PTH ratio >1.5 [11]
	*Renal patients*
	– PTH levels of 51 to 300 pg/mL with normal or decreased calcium levels [18]

Partially functional	*Symptomatic nonrenal patients*
	– On calcium supplementation, with or without vitamin D supplementation
	– Hypocalcemic with normal PTH levels and/or grafted-to-nongrafted arm, PTH ratio >1.5 [11]
	*Renal patients*
	– PTH levels of 21 to 50 pg/mL with normal or decreased calcium levels [18]

Nonfunctional	*Symptomatic nonrenal patients*
	– On calcium supplementation, with or without vitamin D supplementation
	– Hypocalcemic with low PTH levels and/or grafted-to-nongrafted arm, PTH ratio <1.5 [11]
	*Renal Patients*
	– PTH levels <20 pg/mL with normal or decreased calcium levels [18]

PTH: parathyroid hormone.
